# *Trypanosoma cruzi* transmission in a Colombian Caribbean region suggests that secondary vectors play an important epidemiological role

**DOI:** 10.1186/1756-3305-7-381

**Published:** 2014-08-20

**Authors:** Omar Cantillo-Barraza, Duverney Chaverra, Paula Marcet, Sair Arboleda-Sánchez, Omar Triana-Chávez

**Affiliations:** Grupo Biología y Control de Enfermedades Infecciosas (BCEI), Sede de Investigación Universitaria, Universidad de Antioquia, Medellín, Colombia; Division of Parasitic Diseases and Malaria, Entomology Branch, Centers for Disease Control and Prevention (CDC), Atlanta, Georgia USA

**Keywords:** Colombia, Chagas disease, Non-domiciliated triatomines, Epidemiology

## Abstract

**Background:**

Colombia, as part of The Andean Countries Initiative has given priority to triatomine control programs to eliminate primary (domiciliated) vector species such as *Rhodnius prolixus* and *Triatoma dimidiata*. However, recent events of *Trypanosoma cruzi* transmission in localities where *R. prolixus* and *T. dimidiata* are not present suggest that other species are involved in the *T. cruzi* transmission cycle.

**Methods:**

We studied *T. cruzi* transmission on Margarita Island, located on the Magdalena River in the Colombian Caribbean region, where a high number of non-domiciliated triatomines infected with *T. cruzi* inside human dwellings have been observed. A cross-sectional survey including serological studies in humans and parasitological and molecular methods in vectors and reservoirs was conducted. We investigated risk factors for human infection and house infestation, and evaluated the association between abundance of wild triatomines in palm trees (*Attalea butyracea*) across municipalities, seasons and anthropogenic land use*.*

**Results:**

The *T. cruzi* seroprevalence rate in humans was 1.7% (13/743) and autochthonous active *T. cruzi* transmission was detected. The infection risk was associated with the capture of triatomines in human dwellings. Five wild mammal species were infected with *T. cruzi,* where *Didelphis marsupialis* was the main reservoir host with an 86.3% (19/22) infection rate. TcIb was the only genotype present among vectors. Triatomine abundance was significantly higher in Ecosystem 2, as well as in the dry season. Despite the absence of triatomine domiciliation in this area, *T. cruzi* active transmission was registered with a human seroprevalence rate similar to that reported in areas with domesticated *R. prolixus.*

**Conclusions:**

This study illustrates the importance of secondary and household invading triatomines in Chagas disease epidemiology in the Caribbean lowlands of Colombia.

## Background

The success of Chagas disease control programs has been reflected in the significant reduction of *Trypanosoma cruzi* prevalence and disease incidence in several countries of South America [1–3] as well as the interruption of vector-mediated transmission by targeted vector species [4]. However, re-infestation of treated households by native vectors, reemergence of infections through extradomiciliary vectorial transmission, and domiciliary or peridomestic transmission by non-domiciliated vectors, remains a constant challenge for public health authorities [3, 5]. This challenging epidemiological scenario demands the application of alternative strategies, directed toward reducing triatomine entry into houses, such as screens or insecticide-impregnated curtains [6, 7]. The optimization of these strategies demands significant knowledge of the eco-epidemiological features of different transmission foci and the environmental conditions that promote the contact between humans and adventitious household-invading triatomines [2, 8].

Colombia, as part of The Andean Countries Initiative (ACI) has given priority to triatomine control programs to eliminate primary (domiciliated) vector species such as *Rhodnius prolixus* and *Triatoma dimidiata*[9, 10]. However, recent events of *T. cruzi* transmission in localities where *R. prolixus* and *T. dimidiata* are not present suggest that other species are involved in the *T. cruzi* transmission cycle [11]. In Colombia, 24 triatomine species other than *R. prolixus* and *T. dimidiata* have been reported, 15 of which are naturally infected with *T. cruzi*[12, 13]. The eco-epidemiological features related to non-domiciliated vector *T. cruzi* transmission, including infection rates and the specific factors affecting transmission in areas where these triatomine vector species are present remain unknown.

In Colombia’s lowland Caribbean region, *R. pallescens* and *Triatoma maculata* are the most important Chagas disease vectors [12]. *R. pallescens* is closely associated with the palm tree *Attalea butyracea*, where it maintains an enzootic *T. cruzi* cycle [12, 14]. Moreover, it is a very important *T. cruzi* vector across regions, responsible for most of the *T. cruzi* transmission to humans and domestic animals in countries such as Panama and Costa Rica [15–17]. The environmental variables associated with this species and its increasing contact with human hosts have been thoroughly studied [16, 18]. Nevertheless, this relationship has not been evaluated in Colombia’s lowland Caribbean region, where the presence of agriculture and livestock increases *A. butyracea* density near human dwellings.

*T. maculata* is considered in the domiciliation process in the lowland Caribbean region [12]. The epidemiological role for this species is not yet clear because it is usually reported without natural *T. cruzi* infection and there are no reports of active human transmission in areas where *T. maculata* is the only triatomine recorded [12, 19]. However, in Talaigua Nuevo (Bolivar Department, Colombia), *T. maculata* has been reported to harbor natural *T. cruzi* infection in an active transmission focus area [20]. Hence, more expansive epidemiological studies are necessary to determine the actual burden and implication of this species as a Chagas disease vector in Colombia.

On Isla Margarita (Margarita Island), located in the Caribbean lowlands of Colombia, Cantillo *et al.*[21] reported four triatomine species with high *T. cruzi* infection rates: the mostly sylvatic *Rhodnius pallescens*, *Eratyrus cuspidatus* and *T. dimidiata* and the peridomestic *T. maculata*. All four species had occasionally been reported invading houses. Moreover, *T. cruzi* transmission was previously reported in this region, suggesting that vectorial transmission could be occurring through (secondary) non-domiciliated vectors in this part of Colombia [20]. However, the characterization of this epidemiological scenario is needed so that all components of the transmission cycle can be considered and evaluated. Therefore, the aims of the present study were to: a) evaluate the seroprevalence of *T. cruzi* in the human population of Margarita Island, b) describe the environmental factors that could be associated to sylvatic triatomine abundance, c) establish the dwelling features that are related to triatomine infestation, d) identify the main *T. cruzi* reservoirs and e) determine the *T. cruzi* genotypes present in the region.

## Methods

### Study area

**Figure 1:**
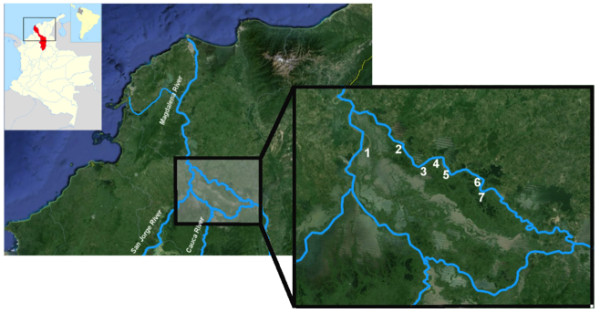
**Margarita Island is shown on a Google Earth image; it is a portion of land surrounded by arms of the Magdalena River, located in the Colombian lowland Caribbean region (Bolivar Department indicated in red).** Sampling municipalities are indicated as 1. Cicuco, 2. Talaigua Nuevo, 3. La Rinconada, 4. Tierrafirme, 5. Mompos, 6. San Fernando and 7. Margarita.

This study was carried out on Margarita Island, located in the Department of Bolivar in Colombia’s Caribbean Region. The island has an area of 2930 km^2^ with an altitude of 30 m a.s.l. It is the largest flooding zone in Colombia. The study area was extensively described in Cantillo *et al.*[21]. Briefly, it is a tropical subhumid region with an annual rainfall of 1660 mm and an annual average temperature of 28°C. Rainy seasons are bimodal extending from April to June and August to November. The dry season extends from December to March.The survey was carried out in five municipalities on the island: Mompos (including the rural areas Tierrafirme and La Rinconada), Talaigua Nuevo, Cicuco, San Fernando and Margarita (Figure 1).

### Sample size and randomization of houses

We carried out a cross-sectional study, where the number of houses included in the study was equally represented, according to the information available in the census conducted by The Colombian Government Demography Agency, DANE, Colombia, in 2006 [22]. Margarita Island has approximately 17.166 houses. For selection of houses, first we made a random selection of block in each population and after we randomized houses using a computer list of random numbers. To estimate the appropriate and representative sample size (*n*) the Epi-info V 2000 software was used. A 4% confidence level and 50% frequency of infestation (because prevalence is unknown) were used. One sample of 580 houses that represented the proportion of houses of each municipality was obtained and distributed as follows: Mompos, 282 (48.8%); Talaigua Nuevo, 74 (12.7%); Cicuco, 75 (12.9%); San Fernando, 74 (12.7%) and Margarita, 75 (12.9%).

### Epidemiology survey

One person for each family living in studied houses was selected randomly by choosing a red ballot. This procedure was done until the sample was complete. We calculated one representative sample based on information available in Colombian Government Demography Agency DANE [22]. Margarita Island had approximately 85.000 inhabitants in 2006. To estimate the appropriate and representative sample size (*n*) for the human survey, the Epi-info V 2000 software was used. A 5% confidence level and 50% frequency of infection (because prevalence is unknown) were used. One sample size of 596 people was calculated. However, this was increased by 25% due to the high mobility of the population in the study area during the flooding period. Four inclusion criteria were defined for ensuring that participants could have become infected in this area: 1) the person must have been born in and lived their entire life in the study area, 2) we verified that randomization of participants was made on people that live in the house that was being evaluated, 3) the participants had not received blood transfusion, and 4) we did not include any person with a previous diagnosis of Chagas disease or leishmaniasis. All who agreed to participate signed a written consent form. For children, one parent signed the form, following the requirements of the University of Antioquia Ethics Committee (protocol number 05-041-005/2005).

### Analytical approaches

To identify the risk factors associated with *T. cruzi* infection and infestation (defined as the presence of insects bugs in home) we used the standardized national cross-sectional survey of Chagas disease risk in Colombia [9, 23]. This survey includes socioeconomic factors recognized as relevant for the risk of infection and infestation such as housing conditions, history of contact with the triatomine vector, ability to identify the vector, resident reports of triatomine sightings in different areas of the house, time since the last insecticide spraying of the house, presence or absence of animals in the house and capture of triatomine bugs in the house. The epidemiological questionnaire was given to the head of each family. The house infestation data used for the analysis was obtained in the entomological survey reported in Cantillo *et al.*[21]. Data management and statistical analyses were conducted using SPSS V.15.0 and Epi-info V 2000. A primary screening (univariate analysis) was performed using 2 × 2 contingency tables using as dependent variables infestation and infected individuals with exposure variables. All variables with *p* < 0.1 and eight variables with traditional importance in Chagas disease epidemiology were analyzed by logistic regression, using SPSS. The logistic regression was used to control for possible confounding variables [24]. The best fit model for both categories was selected on the basis of its Negelkerke R square value.

### Serological methods

Approximately 5 mL of whole blood was collected from each patient by venipuncture. The samples were centrifuged and the serum was stored at −30°C until processing. Two serological tests were applied to detect anti-*T. cruzi* IgG. All samples were subjected to one initial ELISA (enzyme-linked immunosorbent assay) homemade screening test, using total protein extract as antigens, prepared from *T. cruzi* isolates (I.RHO/CO/00/CAS-15.CAS; I.TRI/CO/03/MG-8.MAG). Previously confirmed positive and negative samples were used as control values of optical density (OD) to define the limits for seropositivity and seronegativity. OD values higher than 2 SD from the average OD for negative controls were considered seropositive. All positive ELISA samples were confirmed by an indirect immunofluorescence antibody test (IFAT) with a titer of 1:40 as the positive cut-off. Samples that were seropositive with both tests were considered seropositive for *T. cruzi*.

### Entomological analyses

Palm trees (*n* = 50) (*A. butyracea)* located near households were examined for triatomines following the methodology described in [25]. Dry and green leaves, organic debris, interfoliaceus meshes and bracts were examined for the presence of triatomines. The statistical association between abundance of *R. pallescens*, *E. cuspidatus* and habitat, seasonality (rainy or dry season) and locality were studied. Habitat types were classified according to vegetation height and composition surrounding the palm trees around a 100-m^2^ area. Four habitat types were defined according to a scale based on land use and vegetation coverage [18]: a) Ecosystem 4: areas of abandoned pastures or cropland undergoing forest succession, covered with opportunistic vegetation, on average above 2 m in height with trees higher than 5 m; b) Ecosystem 3: abandoned pastures with opportunistic vegetation 1–2 m high, bushes, fruit trees and the presence of lianas; c) Ecosystem 2: cattle pastures with the presence of small bushes and opportunistic vegetation; and d) Ecosystem 1: cattle pastures without opportunistic vegetation.

We used Generalized Linear Models (GLMs) with a log link to test the associations among environmental factors as (habitat types, seasonality and locality), and the different combinations of these factors with adult and nymph abundance. The most parsimonious model was identified using the value of Akaike’s Information Criterion (AIC) [26].

### Wild host survey and detection of *Trypanosoma cruzi*in small mammals

Small mammals were captured using traps (Tomahawk® and Sherman®) baited with a mixture of peanuts, bananas, oats and fish. At each locality the traps were set for three nights in the forests where palms were sampled and were distributed in linear transects, with capture points established 20 m apart. Additionally, mammals present in the palms during wild triatomine collection were captured as were mammals found while actively searching for triatomine bugs in human households [21]. For *T. cruzi* diagnosis in nonhuman vertebrate hosts*,* xenodiagnostic tests were performed with 10 *R. prolixus* 5^th^ instar nymphs. Mammals were anesthetized (50 mg/kg body weight of ketamine, administered by intramuscular injection) and insects were allowed to feed on the animal for 20 min. *R. prolixus* feces were later examined for moving parasites, four times within 60 days post-feeding using direct microscope observation at × 400. DNA was extracted using the phenol-chloroform method [27] and *T. cruzi* infection was confirmed by PCR amplification of *T. cruzi* satellite DNA [28].

### Isolation, culturing and genetic characterization of *Trypanosoma cruzi*

Samples of the intestinal content of *R. pallescens* captured in palm trees in each of the five municipalities were macerated in sterile PBS (pH 7.2) and examined microscopically as wet smears (×400). Fecal material from positive triatomines was inoculated subcutaneously into a group of three BalbC mice raised in our laboratory. Tail blood samples were examined 3 days after inoculation and three times weekly thereafter until flagellate forms were seen. Blood parasites were cultured in Novy-McNeal-Nicole (NNN) medium with liver infusion tryptose medium (LIT) overlay. Total DNA was extracted from each culture using the phenol-chloroform method and *T. cruzi* presence was confirmed by satellite DNA PCR amplification [28]. Positive *T. cruzi* samples were then submitted for molecular discrimination of *T. cruzi* DTUs based on the amplification and sequencing of the intergenic spacer of spliced-leader gene (SL-IR) amplified with primers TCC-(5′-CCC CCT CCC AGG CCA CAC ACTG-3′), TC1 (5′-GTGTCCGCCACCTCCTTCGGGCC-3′) and TC2 (5′-CGTACCAATATAGTACAGAAACTG-3′) as previously reported [29]. Amplification products were run on a 1.5% agarose gel stained by ethidium bromide and visualized under UV light. Successful PCR products were purified using a multiscreen plate purification kit (Millipore). DNA was sequenced in both forward and reverse directions with an ABI 3500 (Applied Biosystems) automated sequencer. Sequences were assembled and reconciled using DNA Star Seqman Pro (DNASTAR, Inc., Madison, WI, USA). Reference sequences for each TcI genotype described were retrieved from Genbank: TcIa SN8cl1 (**EU127305),** TcIb FChC (**AM259469),** TcIc EFC (**AM259474)** and TcId PALC (**AM259473)**. Due to the reported presence of INDELs at the microsatellite region (positions 14–40) and the possibility of detecting multiple ambiguous alignments [30, 31], we performed global pairwise alignments by measuring the higher sequence identity between our samples and the reference sequences.

## Results

**Figure 2:**
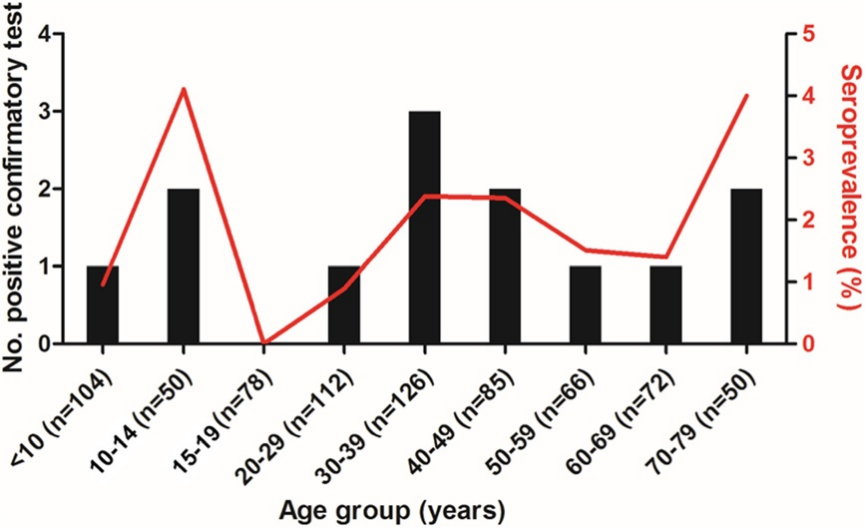
**Prevalence of** ***T. cruzi*** **infection by age group among habitants of municipalities sampled on Margarita Island.** The number of people sampled in each age group is shown in parentheses.

We collected a total of 743 human blood samples in 560 randomized houses. The sera samples were proportionally distributed and collected from patients ranging from 1 to 91 years of age, of which 66% were women and 34% men. The number of samples analyzed per house varied between 1 and 8 with an average of 1.32 sampled per house. 28.6% of the samples were represented by under-18-year olds. A total of 13 patients were positive for two serological tests corresponding to an overall seroprevalence of 1.7% (95% CI = 0.68–2.15%). There was no significant sex bias on infection rates. The results of serological tests were immediately given to each adult or to the person legally in charge of the minors. Adults and children were referred to the local hospital and their results were remitted to Bolivar Departmental Health Secretary (BDHS) as well as their social security (EPS) with the aim of therapeutic management and clinical surveillance.The seroreactivity by age group is presented in Figure 2. The highest seroprevalence was found in the 10- to 14 years old age group (4.1%). However, we did not find a statistical difference between age groups. In the under-15-year old age group, two children from Talaigua Nuevo and one child in rural Mompos were positive. The prevalence of infection within this class was 1.95% (3/154). In order to discard, other transmission pathways, we evaluated the infection status of the mothers of seropositive children and none of them were positive. Moreover, a second visit was made to each house where seropositive people were living to moniter any outbreaks with fever episodes following collective feeding. We did not find any evidence about outbreak of acute Chagas disease associated with oral transmission.

The Talaigua Nuevo and rural Mompos municipalities presented the highest *T. cruzi* prevalence: 4.2% (4/94) and 3.05% (7/229), respectively. San Fernando and Margarita had just one seropositive individual each, presenting 0.96% (1/103) and 1.15% (1/86) seroprevalence, respectively. No statistical differences were found between these municipalities. No seropositive cases were found in urban Mompos and Cicuco.

### Risk factor analysis for *Trypanosoma cruzi*infection

The univariate analysis used to identify risk factors associated with human infection determined the following significant factors: presence of triatomines inside human dwellings [OR 37.22; 95% CI (9.5–150.4; *p* = 0.0001)] and houses with roofs made of palm leaves [OR 7.95; 95% CI (1.65–52.32; *p* = 0.003)]. However, the only factor that remained significant in the multivariate model was the presence of triatomines within houses [OR: 50.429, 95% CI (13.029–182.45; *p* = 0.000)].

### Rate and risk factors for infestation

The infestation rate was 5%. Forty-one triatomine bugs – 39 *T. maculata* and 2 *R. pallescens* – were collected indoors and outdoors (peridomestic area) (Table 1). We did not find evidence about domiciliation indoors as only adults were captured. However, five *T. maculata* nymphs were captured in chicken-coops. The infestation of triatomines was associated with flooring construction material [OR 1.78; 95% CI (0.97–3.26; *p* = 0.045)] and locality. Differences between urban and rural areas within Mompos were observed [OR 8.74; 95% CI (1.97–54.86; *p* = 0.0018)], as well as between urban Mompos and Talaigua Nuevo (OR 22.08; 95% CI (5.01–143.8; *p* = 0.002). Two variables were significantly associated with infestation after adjusting for other possible confounding factors: cement/asbestos/zinc roofs (OR 0.225, 95% CI (0.075–0.826; *p* = 0.007) and locality (OR 0.660, 95% CI (0.075–0.826; *p* = 0.000), both as protection factor against triatomine infestation.

**Table 1:** **Houses in domestic and peridomestic areas infested by secondary vectors (** ***Triatoma maculata*** **and** ***Rhodnius pallescens*** **) on Margarita Island**

Municipalities (Map number)	Houses evaluated (House with Triatomines)	*Triatoma maculata*	*Rhodnius pallescens*
		Adults (Nymphs)	Adults (Nymphs)
Cicuco (1)	72 (0)	0	0
Talaigua Nuevo (2)	74 (12)	12 (2)	1(0)
Mompos (Rural) (3,4)	140 (15)	21 (2)	1(0)
Mompos (Urban) (5)	130 (1)	1 (1)	0
San Fernando (6)	72 (0)	0	0
Margarita (7)	72 (0)	0	0
TOTAL	560 (28)	34 (5)	2 (0)

### Palm tree infestation with wild triatomine bugs

We recovered wild triatomines from 80% (40/50) of the palms analyzed across all habitats and municipalities (Table 2). The lowest proportion of palms infested was in Ecosystem 1 (E1) (33%), followed by Ecosystem 3 (E3) with 90%. In contrast, in Ecosystem 2 (E2) and Ecosystem 4 (E4), all palms were infested. Palm tree infestation was lesser in Cicuco (54%), while in other municipalities the infestation value was higher than 88%.

**Table 2:** **Abundance of wild triatomines in five municipalities on Margarita Island**

Municipalities	Palm trees sample	Infested Palm trees index (%)	*Rhodnius pallescens*		*Eratyrus cuspidatus*		Other species		Density of triatomines by palm tree (SD)
			Adults	Nymphs	Adults	Nymphs	Adults	Nymphs	
Mompos Rural	10	9/10 (90)	17	236	0	0	0	0	25.3 (35.0)
Talaigua Nuevo	9	9/9 (100)	42	197	3	7	0	0	27.6 (28.0)
Cicuco	13	6/13 (46.1)	0	40	0	0	0	0	3.3 (4.9)
San Fernando	10	9/10 (90)	31	348	4	15	0	1^a^	39.9 (40.5)
Margarita	8	7/8 (87.5)	13	141	2	11	1	1^b^	21.1 (30.2)
TOTAL	50	40/50 (80)	1066		42		3		

Four species were found: *Rhodnius pallescens*, *Eratyrus cuspidatus*, ^a^ *Triatoma dimidiata* and ^b^ *Cavernicola pilossa*.

### Factors influencing wild triatomine abundance

GLM analysis revealed that habitat types and season were significantly associated with the overall abundance of triatomines in palms tree (*P < 0.05*). Likewise, *R. pallescens* adult and nymph abundance were associated with habitat types (*P < 0.05*), seasons (*P < 0.05*) and municipalities (*P < 0.05*). With respect to *R. pallescens*, we found that habitat types and season affected the abundance of this species (*P < 0.05*). On the other hand, no differences were found between *E. cuspidatus* abundances and these features.

### *Trypanosoma cruzi*infection in mammal hosts

Twenty-seven mammals were captured: (22) *Didelphis marsupialis*, (1) *Marmosa cf. robinsoni* (Didelphidae), (1) *Diplomys* (Echimyidae), (1) *Oecomis* sp. (Cricetida), (1) *Zygodontomys brunneus* (Cricetidae) and (1) *Rattus rattus* (Muridae). All rodents and five specimens of *D. marsupialis* were captured in palm tree areas. One *T. cruzi*-positive *D. marsupialis* was captured inside a house in Tierrafirme (Mompos rural area). Xenodiagnosis determined high levels of infection, showing *T. cruzi* infection in all species except in *R. rattus* (Table 3).

**Table 3:** **Infection ratio of host species by** ***T. cruzi***

Species	Individuals captured	Municipality	Infection ratio (%)
*Didelphis marsupialis*	22	Mompos (21)	19/22 (86.3)
		Cicuco (1)	
*Marmosa cf. Robinsoni*	1	San Fernando	1/1 (100)
*Diplomys sp*	1	Margarita	1/1 (100)
*Oecomys sp.*	1	Mompos	1/1 (100)
*Zygodontomys brunneus*	1	Cicuco	1/1 (100)
*Rattus tattus*	1	Cicuco	0/1 (0)

Capture locality of each species is shown.

### Molecular characterization of *Trypanosoma cruzi*isolates

Five *T. cruzi* stocks isolated from *R. pallescens* from each municipality in the study area were obtained. They were called *Mom*, *Tal*, *Sfer*, *Marg* and *Cic.* The characteristic 350-bp product for the miniexon SL-region gene (spliced leader region) of TcI was obtained in the five stocks isolated, which showed the highest similarity to TcIb FChC (AM259467), with a sequence identity between 82.1% and 92%. We made hemocultures of seropositive patients but these were negative after three months, therefore, no comparisons could be made with wild-triatomine isolated *T. cruzi*.

## Discussion

In 2010, The National Health Ministry implemented the Colombian Chagas Disease Control and Prevention Program under the Andean Countries Initiative (ACI) (http://www.minsalud.gov.co). The development of these policies has succeeded in interrupting *T. cruzi* transmission by *R. prolixus* in five municipalities of four departments in Colombia [32]. This is a significant result in the advancement of Chagas disease control in Colombia; nevertheless, the epidemiology of Chagas disease in areas without *R. prolixus*, such as the Colombian Caribbean Region, should also be considered. Hence, we suggest that the control of secondary vectors should be included in national health policies, given that human seroprevalence levels are similar to areas where *R. prolixus* is present.

Epidemiological studies on Chagas disease in Colombia have estimated that approximately 5% of people living in endemic zones are infected with *T. cruzi*[33, 34]. The prevalence is higher in the Arauca, Casanare, Santander, North Santander, Boyaca, Cundinamarca and Meta departments, where the *R. prolixus* and *T. dimidiata* vectors are present and domiciliated [12]. Interestingly, the results of the present study showed a human *T. cruzi* seroprevalence of 1.7%, which is also found in areas of primary vector transmission such as Meta (1.7%) and Cundinamarca (1.9%) [33]. In contrast, in Margarita Island primary vectors were not present in households and we did not find evidence of indoor domiciliation of secondary vectors. In this way, the *T. cruzi* transmission in this area could be related with the intrusion of secondary triatomines to homes. This affirmation is supported by the epidemiological association between seropositivity and house infestation as well as the presence of biological elements necessary for autochthonous *T. cruzi* transmission. Moreover, stringed inclusion criteria for greater certainty of vectorial local transmission and epidemiological research to discard other routes of transmission were also taken in account.

On the other hand, similar seroprevalence has been reported in localities where intruder triatomines have high infection rates but are frequently found inside houses [35]. This study provides evidence of the presence of three non primary vectors in the epidemiology of Chagas disease in this Colombian region. However, future work based in molecular epidemiology must be performed to incriminate the vector in the study area. Below we discussed epidemiological relevance of species as *R. pallescens* and *E. cuspidatus* (species documented) and *T. maculata* (species putative) able to infest peridomestic areas.

### Triatoma maculata

The epidemiological role of this species in Colombia, Venezuela and Brazil is still unclear due to its low infection rate [19, 36, 37]. In this regard, this study provides the following evidence that highlights the epidemiological importance of this vector: (i) household-invading behavior in 12.8% of houses in the Mompos rural area and 6.8% in Talaigua Nuevo, (ii) higher seroprevalence and transmission in children under 15 years of age in areas with household-invading behavior (the Mompos rural area and Talaigua Nuevo), (iii) peridomiciliary infestation greater than 10% and (iv) a positive correlation between the presence of this species in houses with human seropositivity. Additionally, we previously reported a *T. cruzi* infection level of 57.1% of *T. maculata*[21], coupled with recent reports of infection of this species in the Caribbean lowlands [38], which supports the need to include *T. maculata* as a priority species in Chagas disease national vector control programs*.*

### Rhodnius pallescens

The anthropogenic activity and deforested landscapes have been correlated with higher abundance of *R. pallescens* in its natural ecotope [18]. The presence of extensive livestock production in the study area has impacted the landscape diversity and increased *R. pallescens* abundance. Our results suggest that palm trees located in cattle pastures with surrounding vegetation (E2) have the highest abundance of *R. pallescens* than palm trees located in cattle pastures without surrounding vegetation (E1). Similar infestation and *R. pallescens* abundance have been reported in palm trees located in mid-secondary forest (remnants or fragments where forest patches remain after large-scale deforestation of late secondary or mature forest) in Panama [18]. Therefore, the high abundance of this vector could be a consequence of anthropogenic land use by livestock production.

The ecological and morphological features of palm trees have been shown to affect the density and infestation of *Rhodnius* spp. on this natural ecotope [14, 39]. For example, the presence of abundant decaying vegetable matter and epiphytic plants increases the likelihood of infestation by *Rhodnius ecuadoriensis*[40]. Additionally, recent studies on the effect of land use/anthropogenic disturbance surrounding *A. butyracea* showed that disturbed habitats were associated with increased *R. pallescens* abundance compared with undisturbed habitats [18]. However, our study also showed that there could be differences in *R. pallescens* abundance in palm trees located inside anthropogenic disturbed habitat since all the palm trees evaluated here were located in cattle pastures. These differences between the triatomine abundance in *A. butyracea* in cattle pastures could be explained by the diversity of vegetation surrounding the palm tree, which can provide food and shelter for potential triatomine hosts.

*R. pallescens* abundance was significantly higher in the dry than in the rainy season. The same situation has been described for *R. pallescens* in San Onofre (Sucre) in the lowland Caribbean region [25]. For this reason, we propose the implementation of increased surveillance of *R. pallescens* during the dry season. Additionally, this species may fly into houses and contaminate food and beverages, possibly an additional risk. On the other hand, significant differences were found between bug abundance and municipalities. Cicuco was the site with the lowest density. This town is surrounded by numerous water reservoirs and the land is therefore highly exploited for livestock use. In contrast, the majority of palm trees evaluated in San Fernando and Talaigua Nuevo, were located in E2 types habitat.

### Eratyrus cuspidatus

This is another sylvatic triatomine species with epidemiological importance in some parts of the lowland Caribbean region. This mostly wild species was incriminated as a *T. cruzi* vector that reportedly invades human dwellings in the region [20, 41, 42]. Although the natural ecotype of *E. cuspidatus* is still unclear, its presence in *A. butyracea* has been reported in many areas of lowland Caribbean Colombia [21, 41]. Our results showed more abundance of nymphs than adults, suggesting that *A. butyracea* is the principal ecotype for this specie in Margarita Island. We did not find association between environmental variables and abundance of this species. The epidemiological aspects associated with increasing contact between this species and human populations are still unknown*.*

#### Final remarks

Anthropogenic intervention has been hypothesized to increase contact between triatomines and humans [43]; however, the greater abundance of wild triatomines in a particular habitat does not necessarily translate into increased Chagas disease risk for humans because socioeconomic and human behavioral factors are also important to consider [18]. Nevertheless, the risk posed by the ability of bugs to invade human dwellings cannot be neglected, because it increases the chances of vector–human contact, the recovery of interrupted domestic transmission cycles [2, 8, 18, 44–46]. Given that increasing numbers of *R. pallescens* and *E. cuspidatus* have been reported in houses in the study region [20], health policies must include actions to survey and control these vector populations.

On the other hand, species from the genus *Didelphis* are considered the classical and most important reservoir for *T. cruzi* given that they are recognized synanthropic animals and can approach human dwellings [47]. In our study area, *D. marsupialis* was the main reservoir system due to its high transmission potential, as shown by the high prevalence of positive xenodiagnosis and high relative abundance of these species in synanthropic habitats. The high frequency of generalist species such as *D. marsupialis* could be a consequence of reduction or elimination of specialist species habitat or positive selection of generalist species that can adapt and survive in the resulting degraded habitats [48, 49]. The consequence of this process is the increased opportunity for contact among infected triatomine bugs with human and domestic animals [50].

The synanthropic behavior of *D. marsupialis* illustrated one important epidemiological role of this species: the linkage of sylvatic and peridomestic cycles of *T. cruzi* transmission. In the present study, ten opossums were collected in palm trees, one inside a *T. maculata*-infested home and one in a palm tree forest. All of the *T. cruzi* samples obtained from different hosts from each locality were characterized as *T. cruzi* DTU I. This lineage is predominant in northern South America, where it may be associated with sylvatic and domestic transmission cycles in northern South America, as well as less severe human disease [51]. Recent studies of this lineage have shown high diversity within DTU I in association with genotypes of epidemiological groups throughout the continent [52, 53]. Sequence analysis of the isolates showed that all strains were identified as TCIb. This genotype has been found in the Colombian population of *T. cruzi* circulating in sylvatic, peridomestic and domestic cycles [52–55].

## Conclusion

In conclusion, the present results suggest that *T. cruzi* transmission with secondary triatomines is occurring where traditional chemical control and prevention programs are not effective on Margarita Island. Thus, the transmission characteristics on Margarita Island argue for alternative strategies able to sustain effective control of native non-domiciliated species. Therefore, we recommend that new control strategies for this area should be based on: (i) active community participation in an entomological surveillance program, (ii) development of a newer survey for data collection without *R. prolixus*, (iii) characterization of *T. cruzi* dynamic transmission in peridomestic areas by *T. maculata* and identification of natural ecotopes, dispersion areas and feeding sources of this species, (iv) intervention and peridomestic reordering and (v) management of *A. butyracea* palm trees in peridomestic areas.
